# Psychological Resilience in Fibromyalgia: The Impact of Mental Health on Clinical Progression

**DOI:** 10.1192/j.eurpsy.2025.1028

**Published:** 2025-08-26

**Authors:** T. Mutlu Topal, H. Ertekin, H. Reşorlu

**Affiliations:** 1 Psychiatry; 2 Physical Therapy and Rehabilitation, Çanakkale Onsekiz Mart University, Çanakkale, Türkiye

## Abstract

**Introduction:**

Fibromyalgia (FM) is a chronic condition characterized by widespread muscle pain,with an unclear etiology. Pain-related behaviors are often closely tied to the individual’s mental state. This has made FM a significant area of psychiatric research, highlighting the need to understand the psychological factors that influence disease progression.

**Objectives:**

We aim to explore differences in psychological resilience between FM patients and healthy controls and its relationship with depression,anxiety,and fibromyalgia symptoms. Specifically,we seek to understand how resilience may influence disease development and progression. By examining these factors, the study aims to clarify the underlying causes of fibromyalgia,is closely linked to psychiatric factors and remains poorly understood in terms of etiology.

**Methods:**

The study included FM patients treated at the Department of Physical Medicine and Rehabilitation at Çanakkale 18 Mart University and a control group of healthy volunteers matched for demographic factors. Pain intensity was measured using the Visual Analog Scale(VAS) and functional disability was assessed with the Fibromyalgia Impact Questionnaire(FIQ). Participants completed sociodemographic forms, the Beck Anxiety Inventory(BAI), the Beck Depression Inventory(BDI), and the Psychological Resilience Scale(PRS).

**Results:**

The study involved 40 female patients with FM and 35 healthy controls. No significant differences were found between the two groups in terms of demographic factors. The FM group showed significantly higher scores for BDI,BAI and PRS compared to controls (p<0.001 for BDI and BAI; p =0.04 for PRS). Correlation analysis revealed significant negative correlations between pain intensity and both PRS total and PRS dedication scores (r=-0.34, p=0.02; r =-0.35, p =0.02). Additionally,FIQ scores were negatively correlated with PRS dedication scores(r=-0.37, p=0.01). (Table 1).

**Table 1: Psychological Status and Performance Scales (BDI, BAI, PRS, etc.)**

**Table:** 

Variable	FM (n, %) & mean ± SD	Control (n, %) & mean ± SD	Statistics	p
**BDI**	20.20 ± 11.54	7.77 ± 6.79	5.58	<0.001
**BAI**	24.90 ± 13.14	9.0 ± 6.97	6.40	<0.001
**PRS Total**	54.92 ± 10.67	59.08 ± 10.27	-1.71	0.09
**Challenge**	21.22 ± 5.82	21.11 ± 5.50	0.08	0.93
**Dedication**	17.75 ± 5.14	19.54 ± 4.24	-1.63	0.10
**Control**	16.25 ± 5.01	18.38 ± 3.70	-2.05	**0.04**

BMI: Body Mass Index, BDI: Beck Depression Inventory, BAI: Beck Anxiety Inventory, PRS: Psychological Resilience Scale

**Conclusions:**

This study highlights the complex interplay between psychological resilience,depression,anxiety and pain in FM patients. Psychological resilience was not significantly lower in FM patients compared to healthy individuals,but higher levels of depression and anxiety were inversely related to resilience.These findings underscore the need for treatment approaches that not only address physical symptoms but also aim to enhance psychological resilience in order to improve the overall quality of life in FM patients.

**Disclosure of Interest:**

None Declared

